# From Omics to Drug Metabolism and High Content Screen of Natural Product in Zebrafish: A New Model for Discovery of Neuroactive Compound

**DOI:** 10.1155/2012/605303

**Published:** 2012-08-05

**Authors:** Ming Wai Hung, Zai Jun Zhang, Shang Li, Benson Lei, Shuai Yuan, Guo Zhen Cui, Pui Man Hoi, Kelvin Chan, Simon Ming Yuen Lee

**Affiliations:** ^1^State Key Laboratory of Quality Research in Chinese Medicine, University of Macau, Avenue Padre Tomás Pereira S.J., Taipa, Macau, China; ^2^Institute of Chinese Medical Sciences, University of Macau, Avenue Padre Tomás Pereira S.J., Taipa, Macau, China; ^3^Faculty of Pharmacy, The University of Sydney, NSW 2006, Australia; ^4^Centre for Complementary Medicine Research, University of Western Sydney, NSW 2560, Australia

## Abstract

The zebrafish (*Danio rerio*) has recently become a common model in the fields of genetics, environmental science, toxicology, and especially drug screening. Zebrafish has emerged as a biomedically relevant model for *in vivo* high content drug screening and the simultaneous determination of multiple efficacy parameters, including behaviour, selectivity, and toxicity in the content of the whole organism. A zebrafish behavioural assay has been demonstrated as a novel, rapid, and high-throughput approach to the discovery of neuroactive, psychoactive, and memory-modulating compounds. Recent studies found a functional similarity of drug metabolism systems in zebrafish and mammals, providing a clue with why some compounds are active in zebrafish *in vivo* but not *in vitro*, as well as providing grounds for the rationales supporting the use of a zebrafish screen to identify prodrugs. Here, we discuss the advantages of the zebrafish model for evaluating drug metabolism and the mode of pharmacological action with the emerging omics approaches. Why this model is suitable for identifying lead compounds from natural products for therapy of disorders with multifactorial etiopathogenesis and imbalance of angiogenesis, such as Parkinson's disease, epilepsy, cardiotoxicity, cerebral hemorrhage, dyslipidemia, and hyperlipidemia, is addressed.

## 1. Introduction

The zebrafish (*Danio rerio*) is a tropical freshwater fish that has become one of the most popular vertebrate model organisms in biological research. The zebrafish has traditionally been used as a model for studying developmental biology and embryology. Recently, zebrafish has become famous in the fields of genetics, environmental science, toxicological studies, and especially drug screening [1, 2].

Zebrafish are small, even adults are only 3-4 cm long, and are suitable, therefore, for animal studies in laboratories with limited space. Their high fecundity enables each adult female to produce hundreds of eggs per mating at intervals of only a few days. The embryos grow and develop rapidly. By 120 h after fertilization (hpf), the heart, liver, brain, pancreas, kidney, and other organs are completely developed. Zebrafish cardiovascular, nervous systems and metabolic pathways are highly similar to those of mammals at the anatomical, physiological, and molecular levels. The zebrafish genome is highly similar to the human genome, with approximately 87% similarity [2]. Their pharmacological response is comparable with that of human, suggesting applications in identifying test compounds with therapeutic potential. The larvae are only 1–4 mm long and can survive in a single well of a standard 384-well plate for several days by using the nutrients stored in the yolk sac. Assay studies require only a small quantity (10–100 ng) of test compounds, such as small molecules, which are easily absorbed through the skin and gills, or directly by swallowing after 72 hpf. Early zebrafish embryos and larvae are optically transparent, which allows real-time imaging* in vivo*. These advantageous features combine and make zebrafish an ideal model for studying the biological activity profiling of natural products containing complex chemical components.

## 2. Relevance and Predictability of Drug Response between Zebrafish and Human

Using zebrafish as a model for drug screening will always raise the question of whether the beneficial effect of a drug lead compound observed in zebrafish would have clinical relevance. Although it has been shown that the zebrafish and human genomes are highly similar, a study should be done to compare the physiological response of human and zebrafish after exposure to a series of drugs. Mittelstadt has tested the effect of nine drugs with QT prolongation in zebrafish and found eight of these compounds (except procainamide) induced dissociation between the atrium and ventricular rates (Table 1) [3]. A similar study was done by Berghmans, who measured the atrial and ventricular rates of zebrafish in response to seven known QT-induced drugs and 2 negative controls and found that 7 of the 9 compounds, including the negative controls, showed the expected effects (Table 1) [1]. Two studies focused on the optomotor response were followed independently by Berghmans and Richards They both found zebrafish showed a high percentage of predictability (~78% and ~70%) of drug response (Table 1) [1, 4]. The zebrafish is also a good model for screening drugs with potential seizure liability. Winter reported the animal model offered 72% overall predictability as 13 out of 17 positive controls and 5 out of 8 negative controls showed their predicted effects (Table 1) [5]. Orally active anti-VEGF agents including sunitinib malate and ZM323881 effectively blocked hypoxia-induced retinal neovascularization in zebrafish. [6]. Two known antiangiogenic compounds, SU5416 and TNP470, which has shown antiangiogenic activity in mammalian system, have also demonstrated reduced vessel formation in zebrafish [7]. A range of known sedative compounds such as clozapine, fluoxetine, melatonin, diazepam, and pentobarbital have comparable response in zebrafish and all of these compounds resulted in reduced locomotor activity. [8–10]. Also, zebrafish also showed comparable responses to toxins for inducing pathologic consequences mimicking Parkinson's disease and epilepsy which will be addressed in later chapters. These evidences suggested that zebrafish demonstrate a good correlation with clinical relevance and support its potential as a model for pharmacological assessment [1, 3–5].

## 3. Similarity of Drug Metabolism between Zebrafish and Mammals: Omics Approach Provides a Clue

It is a common phenomenon that many compounds that occur naturally in metabolic tissues, such as liver and gastrointestinal tract, are inactive *in vitro* but are bioactivated *in vivo* into an active metabolite. One pioneer study with a zebrafish screen searched for cell-cycle modulators and identified 14 active candidates from a library of 2000 compounds (0.7% positive hits). The cell cycle-modulating activities of the active compounds identified from the zebrafish screen were validated in cell lines derived from both zebrafish and mammals [11]. Interestingly, only half of the active compounds were shown to be active in both embryos and either one of the cell lines, showing that some compounds are active in *in vivo* but inactive *in vitro*. The use of zebrafish for drug screening as well as for various pharmacological studies has received increasing attention in the fields of drug absorption, metabolism, distribution, and excretion [12]. However, there are few reports in the literature of detailed systematic studies investigating the fate of drugs after absorption as well as identification of the enzymes involved in drug metabolism in zebrafish larvae.

**Figure 1:**
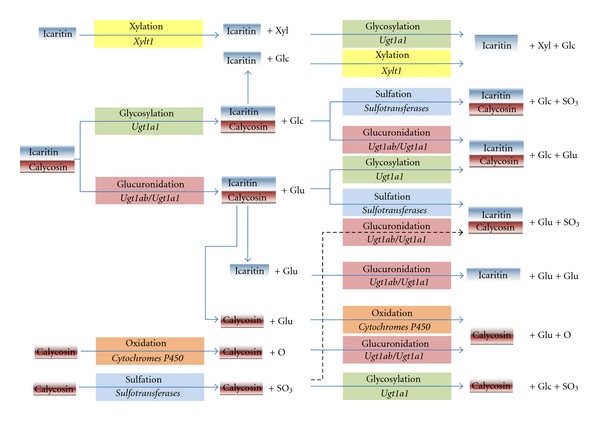
The metabolism of icaritin and calycosin in zebrafish embryos and larvae. The proposed routes of how icaritin and calycosin are metabolized in zebrafish embryos and larvae are summarized and some drug metabolism enzymes are identified by omics approach. The process and the corresponding gene are shown in each colored box. Glc: glycosylated group; Glu: glucuronidated group; Xyl: xylated group; SO_3_: sulfonated group; *Xylt1*: Xylosyltransferase 1; *Ugt1a1*, *Ugt1ab*: UDP-glucuronosyltransferase.

Our recent study addressed drug absorption and metabolism in zebrafish embryos and larvae. We used LC-MS/MS to identify and profile the metabolites of icaritin and its glycoside derivatives in zebrafish larvae [13]. Icaritin is a prenylated flavonoid compound that is regarded as an active ingredient of *Herba Epimedii*, which has been widely used in China as a medicinal herb for the treatment of infertility, osteoporosis, and weakness of the limbs. The result showed clearly that the metabolic pathway involving icaritin and its glycoside derivatives in zebrafish larvae is similar to that reported in mammals (Figure 1). The first step in the pathway is the enzymatic removal of the sugar moiety of these compounds after consumption in the cells of the gastrointestinal mucosa or by enzymes secreted by the colon flora [14]. Hydrolysis of the flavonoid derivative produces the free aglycone, which is conjugated by sulfation, glucuronidation, or methylation or in different combinations with steps that are controlled by phase II metabolism enzymes.

In order to investigate whether zebrafish larvae express the essential drug-metabolizing enzymes that are involved in the proposed metabolic pathways for the production of icaritin and its glycoside derivatives, combined transcriptomic and proteomic approaches were used to identify these enzymes [13]. In fact, transcriptomic profiling procedures identified 51 unique mRNA transcripts (out of a total of 13,310 nonredundant mRNA transcripts) that belong to three categories of key enzymes involved in phase I drug metabolism [15], including the cytochrome P450 family, flavin-containing monooxygenases, and epoxide hydrolases in zebrafish larvae. Moreover, mRNA transcripts of several key phase II drug metabolism enzymes [16], including UDP-glucuronosyltransferase, sulfotransferases, catechol-*O*-methyltransferase, and glutathione-*S*-transferases, were identified. However, the proteomic approach identified only three proteins (out of 2998 distinct proteins) that belong to the glutathione-*S*-transferases, a major type of phase II detoxification enzymes. The result illustrates that the metabolism of icaritin and its derivatives in both zebrafish larvae and mammalian models are highly conserved.

In addition, calycosin, an active constituent in Radix Astragali, was found to promote angiogenesis in zebrafish and human endothelial cells involving activation of the estrogen receptor and mitogen-activated protein kinase (MAPK) signaling pathway [17]. Our recently accepted paper characterizes drug absorption and metabolism using calycosin as a probe in zebrafish larvae [18]. Ten metabolites of calycosin produced by glucuronidation, glycosylation, sulfation, oxidation, or combinations of any two of these metabolisms in zebrafish larvae were identified by LC-MS/MS (Figure 1). The results showed the kinetic changes of calycosin and its metabolites in zebrafish larvae. This study identified drug metabolites previously identified in mammals, reconfirming the conservation of drug metabolism systems in zebrafish and identified novel metabolites, providing insight into the possibility of the discovery of novel drug metabolite diversity in zebrafish. In addition, the abundance of calycosin and its metabolites were increased steadily during 24 h after treatment [18], which reflects the difference of common drug administration between zebrafish and mammals. Unlike the common drug administration routes, such as gastric irrigation and oral administration, used in rodents, the drug treatment for zebrafish was usually performed by keeping the whole fish in a drug-containing incubation medium. This method keeps the zebrafish in an environment of constant drug concentration and drug compounds are continuously taken into the body through both the GI tract and the respiratory systems. Future in-depth systematic investigation of absorption, distribution, metabolism, and excretion (ADME) in zebrafish is warranted.

The high similarity of phase I and phase II metabolisms in zebrafish may be attributed to the highly conserved genetic expression profiles in liver as well as gut microbiota with human and mice counterparts, respectively [19, 20]. Drug screening in other small invertebrate model organisms, such as the fruit fly *Drosophila melanogaster*, has identified some very promising lead compounds, particularly for antiaging [21]. Nonetheless, the proof of concept of the highly conserved drug metabolism between zebrafish and mammals strongly supports the usefulness of zebrafish as a vertebrate model rather than other invertebrate model organisms for drug discovery as well as drug metabolism studies.

## 4. Presence of Blood-Brain Barrier (BBB) in Zebrafish

The BBB is crucial for the maintenance of a stable environment with the regulation of ionic balance and nutrient transport and the blockage of potentially toxic molecules. The intrinsic complexity of the cell-matrix-cell interactions of the neural-vascular unit has made analysis of gene function difficult in cell culture, tissue explants, and even animal models. The zebrafish has emerged as a premier vertebrate model for analyzing the complex cellular interactions *in vivo* and the genetic mechanisms of embryonic development [22]. Brain endothelial cells show immunoreactivity to Claudin-5 and Zonula Occludens-1 (ZO-1), implying the presence of tight junctions in these cells. The expression of Claudin-5 and ZO-1 was detected in cerebral microvessels starting from 3 dpf, concomitant with maturation of the BBB [23]. Zhang et al. observed that zebrafish embryos develop BBB functions by 3 dpf, with earlier expression of Claudin-5 in the central arteries at 2 dpf [24].

Our recent study of the neuroprotective effect of quercetin shed light on the presence of functional BBB in zebrafish larvae at 3 dpf and the role of BBB permeability in determining the beneficial effect of a neuroprotective drug in Parkinson's disease (PD) in *in vivo*. Quercetin is one of the commonest naturally occurring flavonoids. Although it and structurally related flavonoids have been shown to have a neuroprotective capacity in various *in vitro* and *in vivo* experimental models [25–27], the neuroprotective effect of quercetin remains controversial. Nevertheless, quercetin did not protect substantia nigra neurons from an oxidative insult *in vivo*, probably due to inefficiency in passing through the BBB in *in vivo* conditions [28]. There is an urgent need for appropriate *in vivo* studies in order to confirm the neuroprotective effect of quercetin and to identify the reason for the discrepancy between findings* in vitro* and *in vivo*. In order to address this controversy, we administered quercetin at different maturation stages of the BBB in zebrafish and we found it can prevent but not rescue the DA neuronal injury induced by 6-OHDA [28]. When quercetin was administered to zebrafish larvae before 3 dpf when BBB is not well established, it could spread rapidly throughout the brain and exert a protective effect against 6-OHDA toxicity. However, when quercetin was administered to zebrafish after 3 dpf, the matured BBB posed an obstacle to quercetin entering the brain, preventing it from rescuing 6-OHDA insult in dopaminergic (DA) neurons. This result supports earlier findings of the presence of BBB in zebrafish by 72 hpf [23, 24].

## 5. Behaviour Screen in Zebrafish

Zebrafish displays learning, sleeping, drug addiction, and neurobehavioral phenotypes that are quantifiable and comparable with those in human [10, 29]. A zebrafish behavioural assay has been demonstrated as a novel, rapid, and high-throughput approach to the discovery of neuroactive, psychoactive, and memory-modulating compounds [30–32]. In the past, a major obstacle to the discovery of psychoactive drugs was the inability to predict how small molecules will alter complex behaviours. Recently, Rihel et al. reported that the multidimensional nature of zebrafish phenotypes enabled the hierarchical clustering of molecules with comparable effects. This behavioural profiling revealed conserved functions of psychotropic molecules and predicted the mechanism of action of poorly characterized compounds [30]. In addition, Kokel and his colleagues used automated screening assays to evaluate thousands of chemical compounds and found that diverse classes of neuroactive molecules led to distinct patterns of locomotor behaviour. They concluded that a zebrafish behaviour assay can rapidly identify novel psychotropic chemicals and predict their molecular targets [31].

## 6. Zebrafish Bioassay Screening for Selectivity

Toxicity is now the first obstacle to drug development. From 2003–2010, the overall success rate for drugs passing from Phase I to FDA approval was only 9% [33]. A high percentage of drug developments failed at different stages, including animal testing or clinical trial, owing to nontolerated side effects and toxicity. As *in vitro* studies, which are usually cell based or molecular based, such as enzymatic or ligand-binding assays, drug screening with these assays predict the potential therapeutic action toward a specific molecular target and/or cell type; however, hidden toxicity and side effects due to interactions of the drug or its metabolites with other molecular targets, are not fully known.

Recently, a number of drugs were withdrawn from the market due to their human ether-a-gogo-related (hERG) cardiac toxicity [34]. The hERG potassium ion channel has a major role during the repolarization of the cardiac action potential, and the blockade of this ion channel can lead to prolongation of the QT interval, which is closely associated with torsade de pointes, a potentially lethal heart arrhythmia [35]. As a result, hERG (I_Kr_) preclinical safety data are an essential part of any investigation of new drug submissions recommended in the FDA ICH guideline [36]. Zebrafish may present a good alternative model for large-scale screening of drug toxicity on QT prolongation through the ERG channel. hERG and its zebrafish homolog (zERG) have a high degree of similarity as zERG shows 99% conserved amino acid sequence in drug-binding and pore domains with the human ortholog [37]. Inhibition or knockdown of the zERG gene resulted in characteristic arrhythmia with 2 : 1 atrioventricular blockage (2 atrial beats coupled to 1 ventricular beat) [37]. The pharmaceutical industry has changed strategy by prescreening compound libraries for hERG cardiac toxicity before screening for therapeutic targets. According to the ICH S7A guidelines, CNS studies including behavior, learning and memory, neurochemistry, optomotor, and/or electrophysiology examinations are recommended before product approval [38]. Zebrafish may be a good model for the CNS assessment, since the animal possesses matched defined area in brain including hypothalamus and olfactory bulb [39]. The hippocampus was proposed to be located in the lateral zone of the pallium in zebrafish [39, 40]. In addition, important neurotransmitter systems such as the cholinergic, 5-hydroxytryptaminergic, dopaminergic, and noradrenergic pathways are also present in zebrafish brain [41, 42]. Zebrafish also has comparable neurological pharmacological response including locomotor activity [10], circadian pacemaking [43], and drug addiction [44] to human counterpart. These evidences support that zebrafish may be physiologically relevant model for screening out neurotoxic compounds.

Assessment of gastrointestinal complications may also be important during drug development, since the adverse reactions may result in death caused by gastrointestinal bleeding [45]. The zebrafish displayed similar physiology in gastrointestinal system with human. For example, the small intestine is lined with most of the cell types except Paneth cells [46, 47]; the peristalsis is controlled by a pair of smooth muscles and regulated by enteric nervous system [48]. However, it did not have a stomach [49] and a submucosa layer containing connective tissue to separate the epithelium from smooth muscle layer [46]. Moreover, in the study of the effect of 10 known compounds on gastrointestinal contraction in zebrafish, 5 out of 10 compounds showed expected effect [1] (Table 1). The relatively low predictability was due to the low reproducibility of cromakalim, nicotine, and nitrendipine in duplicated experiments [1] (Table 1). Nevertheless, zebrafish still has the potential for predicting adverse effects in gastrointestinal system [1]. There is increasing research on predicting the toxicity of a compound and excluding those compounds predicted to be toxic early in the drug discovery process [50].

Efficacy and toxicity are two important criteria for a drug to be marketed and the zebrafish model allows simultaneous measurement of these two parameters. The survival rate and/or mortality are/is a common and direct parameter used to indicate the toxicity of a compound. The beating heart of the embryo is the golden parameter used to indicate the living status of drug-treated zebrafish embryos. Thus, the lethal toxicity of a compound to zebrafish embryos reflected by the heartbeat rate could be monitored simultaneously with observation of the activity, such as antiangiogenesis, associated with the compound of interest. Moreover, other signs of toxicity, such as delayed development of zebrafish embryos, can be observed from the lower level of pigmentation in body and eyes, larger yolk sac, and shorter trunk in response to drug treatment. For instance, in our on-going screening of antiangiogenesis activities of a series of methoxyflavone derivatives, we identified structural modification in a single chemical group of the same scaffold, which exhibited higher potency of antiangiogenic activity and lower toxicity to zebrafish embryos [51]. This pilot study serves as proof of concept, suggesting the advantage of zebrafish over HUVEC cells as an angiogenic assay is that the zebrafish allows content screening of both activity and *in vivo* toxicity.

**Figure 2:**
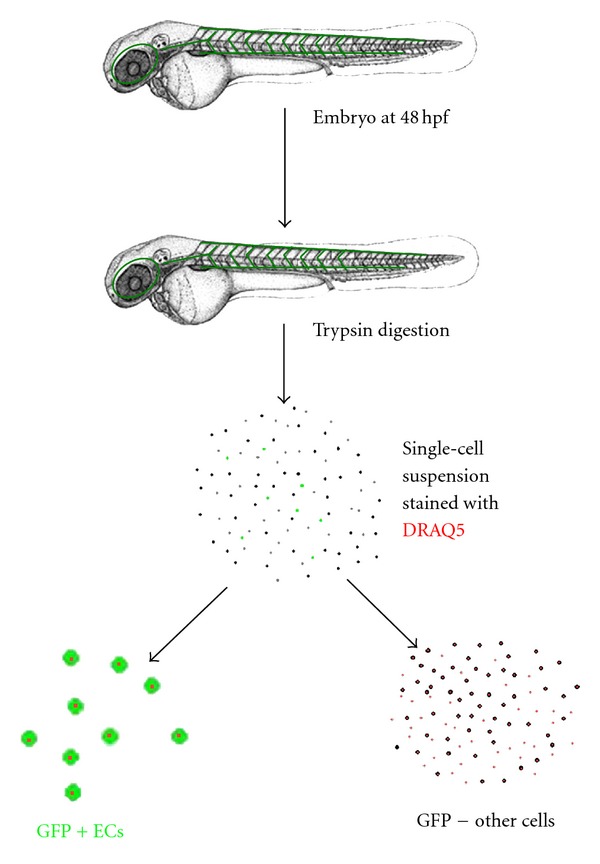
A diagram showing the processing of zebrafish embryos for isolating endothelial cells followed by staining with DRAQ5. Tg(fli-1:EGFP) zebrafish embryos are firstly trypsinized into a cell suspension, stained with DRAQ5, and separated into GFP expressing endothelial cells and others.

**Figure 3:**
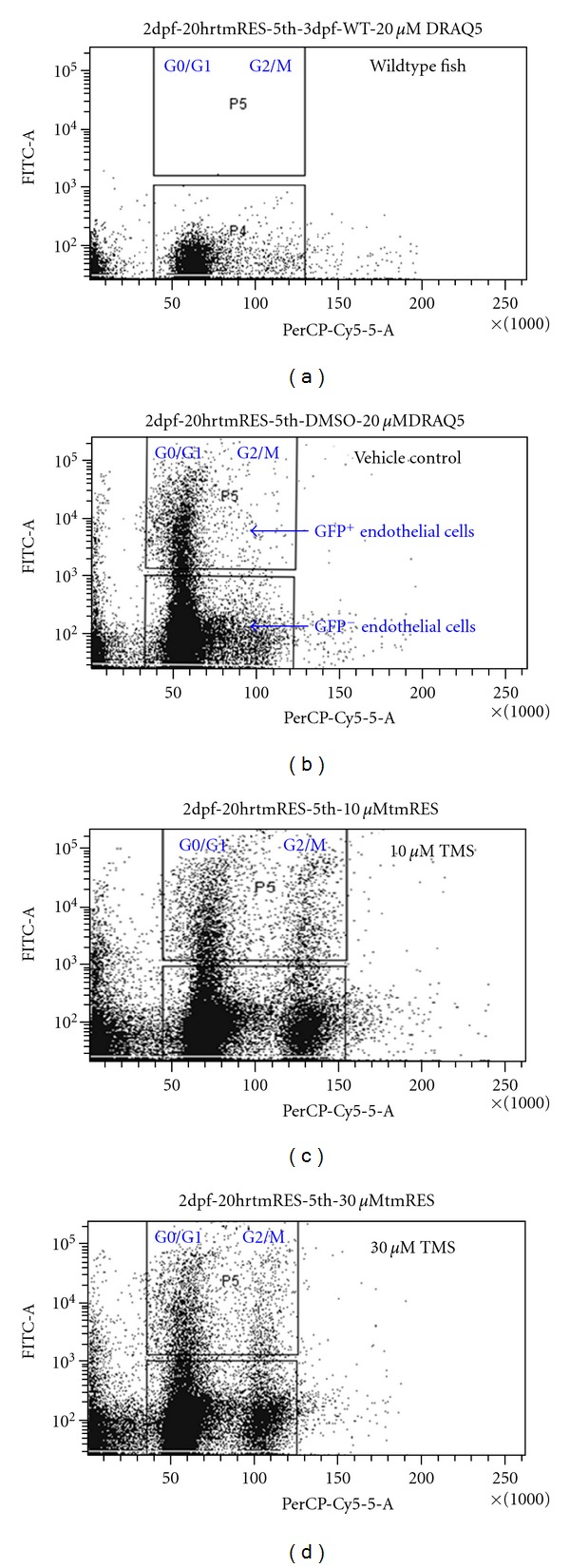
Cell-cycle analysis of zebrafish embryos after treatment with different concentration of an antiangiogenesis compound named trans-3,5,4′-trimethoxystilbene (TMS). Tg(fli-1:EGFP) zebrafish embryos treated with TMS for 20 h were then trypsinized and DRAQ5-stained for cell-cycle studies by flow cytometry. (a) Wildtype embryos did not show GFP-expressing-cells. (b) Tg(fli-1:EGFP) showed GFP expressing and non-GFP expressing cells. (c) 10 *μ*M TMS and (d) 30 *μ*M TMS exerted G2/M cell-cycle arrest preferentially in endothelial cells.

Along with studying an antiangiogenic compound in zebrafish, we could evaluate the selectivity of molecular action, such as cell-cycle arrest, to blood vessel cells in a live organism. Zebrafish embryos were trypsinized into a live cell suspension which was stained with the DNA-staining dye DRAQ5 (Biostatus Ltd., UK) for subsequent cell-cycle analysis (Figure 2). The differential effect of the compound on the cell cycle of endothelial GFP-expressing cells and the non-GFP-expressing cells could be determined by flow cytometry. Using this technique, the resveratrol derivative *trans*-3,5,4′-trimethoxystilbene (TMS) was found to induce cell-cycle arrest more significantly in endothelial cells (in about 20–30% of GFP-positive cells and in only 5–10% of GFP-negative cells) in zebrafish embryos (Figure 3), confirming that TMS exerted a more specific cytotoxic effect on endothelial cells than on other cell types *in vitro* and, more importantly, *in vivo* [52]. However, there was still an overall increase in G2/M phase cells in the whole cell population, indicating that TMS caused cell-cycle arrest in some other cell types (Figure 3). This finding provides a solution to the controversial issue regarding whether resveratrol and related compounds cause cell-cycle arrest through the G1 or G2/M phase in cell culture *in vitro* [53]. This study in zebrafish embryos, which showed the induction of G2/M cell-cycle arrest in GFP-positive endothelial cells by TMS in a whole live organism, provides insight into the physiological relevance of the compound. The concept of determining selectivity of antiangiogenic action on zebrafish endothelial cells was supported by the results of a similar study, in which nobiletin (5,6,7,8,3′,4′-hexamethoxyflavone) exhibited an effect on cell-cycle arrest differently via inducing G0/G1 phase accumulation in GFP-positive endothelial cells [54]. Besides analysis of the cell cycle, a similar approach could probably be adopted to probe cells for different cellular physiological parameters, such as oxidative stress and mitochondrial function, by different stains. This approach allows examination of how a candidate selective drug may act on specific cell types in a live organism.

## 7. Phenomics and Biological Activity Profiling

**Figure 4:**
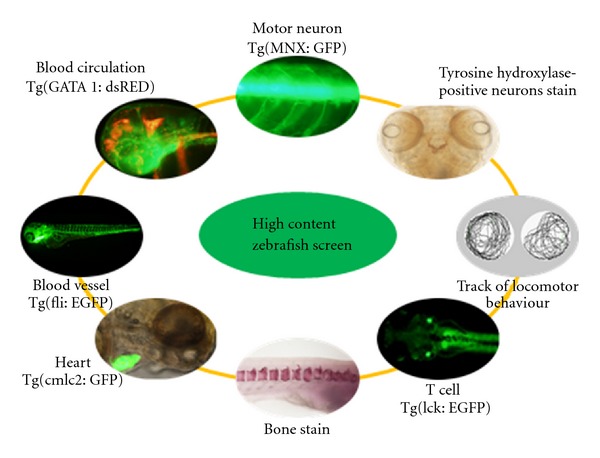
The examples of zebrafish model for high content drug screening. Image-based bioassays reflecting the physiological changes in either wildtype or transgenic zebrafish enable the assessment of multiple pharmacological activities of a chemical compound.

Phenomics was originally an area of biology that involved studies of phenotype as a whole organism. Image-based bioassays reflecting changes of locomotor behaviour in the phenotype of different cell types, organs, and physiological systems in wildtype or transgenic zebrafish offer the opportunity to assess multiple pharmacological activities of a chemical compound (Figure 4). Pharmacological action of a compound could be decoded by a system biology approach through data mining of the multidimensional phenotypic data of an organism [55] together with measurement of the relative levels of mRNA transcripts (transcriptome), proteins (proteome), and metabolite components (metabolome). Recently, this omics approach has been incorporated increasingly into drug discovery and toxicology. Omics data provide much more information than typical phenotypic assay, including observable changes of morphology in the embryo as well as behaviour and mortality. By coupling omics data with an existing phenotypic end-point assay, more details of the mechanism and the toxicity of a chemical could be used to explain the cause-and-effect relationship. Even though the phenotypic changes are the same, such as vitellogenin, which indicate the estrogenic exposure in fish was upregulated by 17b-estradiol, bisphenol A, and genistein [56], the modes of action may differ. This possibility can be shown by differential gene expression induced by these chemicals based on transcriptome analysis [56].

## 8. Integrative Transcriptomic and Proteomic Analysis of Zebrafish

Because the therapeutic action of a drug on normalizing pathological change can originate from different cellular pathways in the complex regulatory network, an *in vivo* study could provide considerably more information than *in vitro* assays using purified targets. mRNA transcripts and proteins are the primary molecules responsible for biological functions in cells and the ability to examine the transcriptome and the proteome of an organism provides a robust overview of the physiological changes taking place and could greatly augment target-oriented biological data. Therefore, we recently used an RNA-seq technology for transcriptome profiling and a fully automatable RP-RP 2DLC system for shotgun proteomics to address the drug metabolism system of zebrafish and the downstream transcriptional effect of a drug. The recent advancement of deep sequencing and 2D RP-RP LC-MS/MS technology identified a total of 12,560 mRNA transcripts (obtained from about 5 million reads per RNA sample) with matched annotated genes [13, 57] and 1752 unique proteins from the zebrafish lysate, respectively, in a single analysis [58].

The current transcriptome profiling tools used in zebrafish are microarray and RNA-seq. RNA-seq or deep sequencing of RNA samples using the next generation of sequencing technology is becoming a popular transcriptome profiling tool because it is an open platform that does not require predefined probes. In principle, RNA-seq profiles known and novel transcripts and it yields data with higher resolution, wider dynamic range, and lower background noise, while it only requires smaller amounts of RNA sample than microarrays [59].

By contrast, proteomic studies have used mainly integrated technologies, including separation of proteins by 2D polyacrylamide gel electrophoresis (2D-PAGE) and identification of proteins by matrix-assisted, laser desorption/ionization time-of-flight mass spectrometry (MALDI-TOF MS). Another major advantage of LC-MS/MS over conventional 2D PAGE is that the high sensitivity of LC allows for faster detection and direct identification of wider range of proteins, including high molecular weight proteins and very acidic or basic proteins, all of which are problematic when using methods such as 2D PAGE [58].

After transcriptomics, proteomics is often considered the next step in the study of biological systems. The integrated study of the two approaches is often considered as a study of causality. The focus is especially on identifying some biological response that initiates at the transcriptional level and exhibits functional information at the protein level. Transcriptomics has advantages over proteomics by allowing larger scale and higher throughput of analysis and about 10 times more coverage of detected gene targets in a single run of a zebrafish sample, while proteomics has advantages over transcriptomics in terms of potentially observing functional change in protein expression and posttranslational levels.

The methodology of conventional immunology depends on the availability of antibodies and *a priori* knowledge of the targets and, in general, is not amenable to global monitoring. Moreover, it is now known that the transcript level is often not correlated with the protein expression level, and the proteomic approach defines responses at the protein level that are probably not regulated at the transcription level, thus providing additional information [60]. Therefore, utilization of integrated transcriptomics and proteomics could provide more confirmatory evidence for the identification of molecular targets involved in the biological response of zebrafish to drug treatment. One of the advantages of integrating transcriptomics and proteomics lies in its neutrality to *a priori* knowledge and targets. The observation-driven result could lead to insights into previously unsuspected targets. The recent advancement of deep sequencing technology and the 2D LC-MS/MS system provides an unprecedented opportunity to formulate a system biology approach to unravel the resulting effect of holistic action of a drug or natural product containing multiple components through either interacting with a specific or multiple targets in a whole organism.

## 9. Zebrafish Disease Models

A highly relevant disease model should be developed when exploring the pathophysiological mechanism and the biological activity of any drug compound. Zebrafish models have been developed in several therapeutic areas for investigating human diseases. Disease models are created by mutation or inactivation of genes, treatment with chemicals, or even modification of a diet.

## 10. Neurodegenerative Disease Models

### 10.1. Parkinson's Disease

PD is the second most common neurodegenerative disease characterized by progressive loss of DA neurons in the substantia nigra pars compacta. The etiology of PD is not completely understood but increasing evidence suggests that oxidative damage induced by reactive oxygen species (ROS) and reactive nitric species (RNS) neuroinflammation, excitotoxicity, and apoptosis are involved in the progression of DA neurodegeneration [61].

Recently, the zebrafish has been demonstrated to be an appropriate model for PD [62]. The DA system in the posterior tuberculum of the ventral diencephalon is comparable with the nigrostriatal system in human [63]. PD-related neurotoxins cause the loss of DA neurons, reduced expression of tyrosine hydroxylase (TH) (Table 2) and the impairment of motor behaviour in zebrafish that are comparable with the pathophysiological features observed in other animal models [64]. In addition, clinical and experimental neuroprotective agents (nomifensine, a DAT inhibitor; L-deprenyl, an MAO-B inhibitor) (Table 2) have been demonstrated to be active in protecting zebrafish from neuronal insult [65]. Either knockdown or mutation of important genes, including PARKIN and LRRK2, contributes to a significant decrease in the number DA neurons (Table 2). Taken together, the results of earlier studies suggest that the zebrafish is a good alternative species for a PD model and offers great opportunity for screening and discovery of novel PD therapeutic agents.

**Figure 5:**
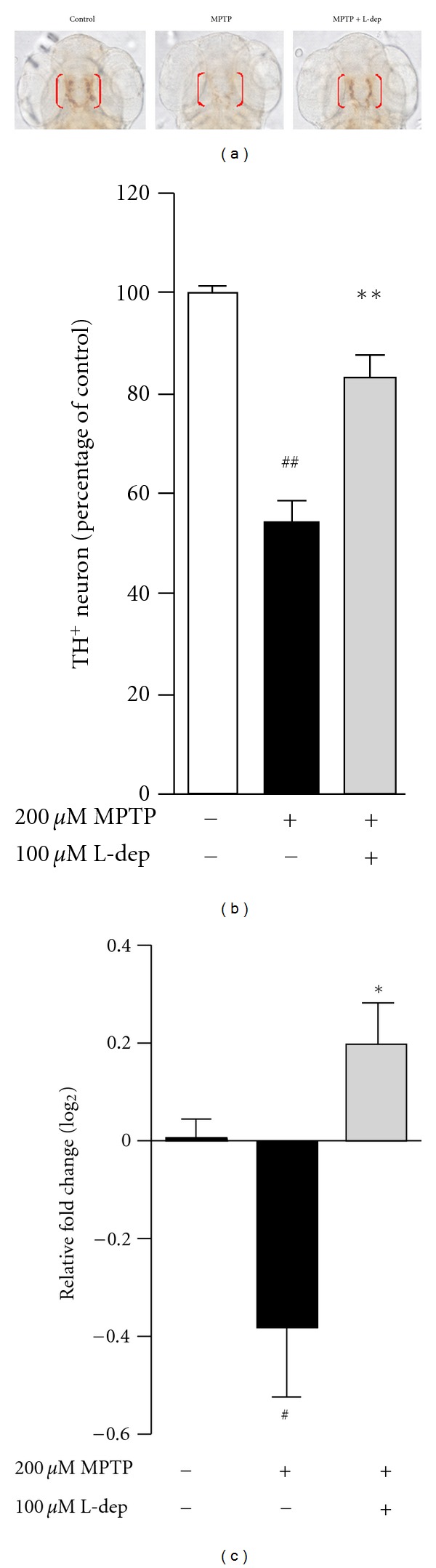
MPTP induces DA neuron loss in zebrafish. (a) Representative picture of anti-TH whole mount immunostaining. TH^+^ neurons in diencephalic region were indicated by bracket, dorsal view. L-dep, L-deprenyl (selegiline), a selective MAO-B inhibitor, was used as positive control. (b) Counting of TH^+^ neuron. (c) Relative fold change of *th* gene expression as compared to control, MPTP downregulated *th* gene expression. ^#^ *P* < 0.05 and ^##^ *P* < 0.01 compared with untreated control. *P < 0.05 and **P < 0.01 compared with MPTP treated alone.

**Figure 6:**
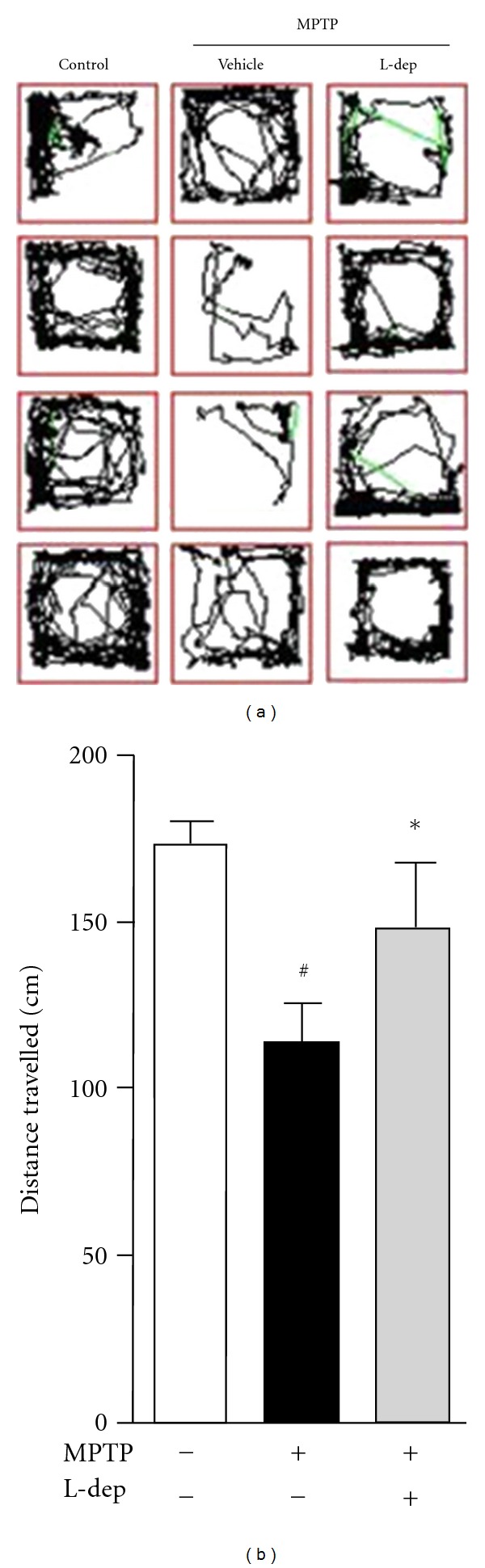
MPTP induces deficit of swimming behavior in zebrafish. (a) Typical swimming patterns of control and MPTP-treated zebrafish. Lines show the track of zebrafish movement. Zebrafish treated with MPTP was less active as compared to the control. (b) Quantitative analysis of total distance travelled. ^#^ *P* < 0.05 compared with untreated control. *P < 0.05 compared with MPTP treated alone.

The brain structure and function of the zebrafish are very similar to those of other vertebrates [70]. The anatomy of the zebrafish brain DA system was studied recently, and a region anatomically similar to the striatum was identified in the forebrain [71]. Neurotoxins, such as MPTP, 6-OHDA, and rotenone, are known to induce DA neuron loss in animal models. Among those neurotoxins, MPTP/MPP^+^ is the best characterized toxin to generate model of PD and has proved useful for studying the striatal circuitry involved in PD pathophysiology [72]. Exposure of zebrafish to MPTP caused profound loss of tyrosine hydroxylase-positive (TH^+^) neurons and downregulated *TH* mRNA expression in contrast to vehicle-treated healthy zebrafish (Figure 5) leading to a deficit in locomotor behaviour (Figure 6). Earlier studies revealed that 6-OHDA is taken up selectively by the plasma membrane dopamine transporter and subsequently accumulates in the mitochondria, resulting in the formation of ROS and RNS [73]. In addition, neuroinflammation plays a key role in 6-OHDA-induced DA neuron damage *in vivo *[74]. We measured the gene expression of proinflammatory mediators in 6-OHDA-treated zebrafish by quantitative real-time PCR and showed that 6-OHDA caused overexpression of* IL-1*β**, *TNF-*α**, and *COX-2*, several-fold higher than that of untreated control fish [75]. These proinflammatory genes play important roles in the etiology of PD [76]. It has been shown that the level of the COX-2 protein is upregulated in substantia nigra DA neurons in PD patients and in animal models [77]. The inhibition of COX-2 and TNF-*α* has provided neuroprotection in rats [76]. Our current iTRAQ-based shotgun proteomics study in a zebrafish model for PD suggested the potential involvement of both TNF-*α*/NF-*κ*B and oxidative phosphorylation pathways in 6-OHDA-induced neurodegeneration in zebrafish (unpublished data). However, given that, all reported promising studies on this chemical induced PD experimental zebrafish model, more researches need to be done to differentiate systemic toxicity and selective neuronal toxicity of the neurotoxins. In addition, generation of transgenic zebrafish expressing fluorescent protein specifically in DA neuron, that allows tracking the kinetic change of living DA neurons *in vivo*, is a viable strategy to replace the postimmunochemical staining of TH-positive neurons.

### 10.2. Epilepsy

Epilepsy is a common neurological disorder characterized by the recurrent appearance of spontaneous seizures due to neuronal hyperactivity, and the disease afflicts nearly 50 million people worldwide [78]. Recent studies showed that the pathogenesis of epilepsy involves altered distribution of GABA receptors (Table 3), enhanced activity of excitatory circuits, neuronal loss, and synaptic reorganization [79–82]. A number of genes encoding transcription factors, synaptic receptors, ion channels, and glucose transporters have shown altered mRNA expression in rodent models of epilepsy (Table 3). These findings suggest potential gene markers other than *c-fos*. Although some antiepileptic drugs (AEDs) are marketed, there is no drug capable of reversing the cause of pathological changes in the brain [83] and some disease subtypes, such as temporal lobe epilepsy, are even resistant to current pharmacotherapies [84]. This problem calls for large-scale screening of new candidates of AED, but this is difficult to achieve in a rodent model.

A PTZ-induced epilepsy model of zebrafish was established by Baraban in 2005 [85], who reported the upregulation of *c-fos* in the CNS region of zebrafish exposed to PTZ. After exposure to PTZ, the larval zebrafish shows three stages of seizure: a dramatic increase in total distance travelled at Stage I, rapid whirlpool-like circling swimming behaviour at Stage II, and culmination in clonus-like convulsions leading to loss of posture at Stage III. Current AEDs can stop the seizure at Stages I and II and, therefore, epileptic zebrafish at both stages are suitable for drug screening [92].

### 10.3. Heart Disease and Cardiotoxicity

Mutations found in cardiac troponin T type 2 (TNNT2) [93] and T-box-5 (Tbx5) are implicated in cardiomyopathy. Severe heart defect was observed in zebrafish carrying the mutated TNNT2. Mutation in Tbx5 leads to the maldevelopment of heart and upper limbs known as Holt-Oram syndrome [94]. Zebrafish carrying the same mutation have comparable deformed heart and pectoral fins [95]. In fact, troponin T was considered as a biomarker in congenital heart failure from dilated cardiomyopathy [96, 97]. Other biomarkers, such as myosin light chain-I [96, 97], cardiotrophin [98], and endothelin-1 [99], are proposed to have diagnostic value in congestive heart failure and hypertension (Table 4).

**Figure 7:**
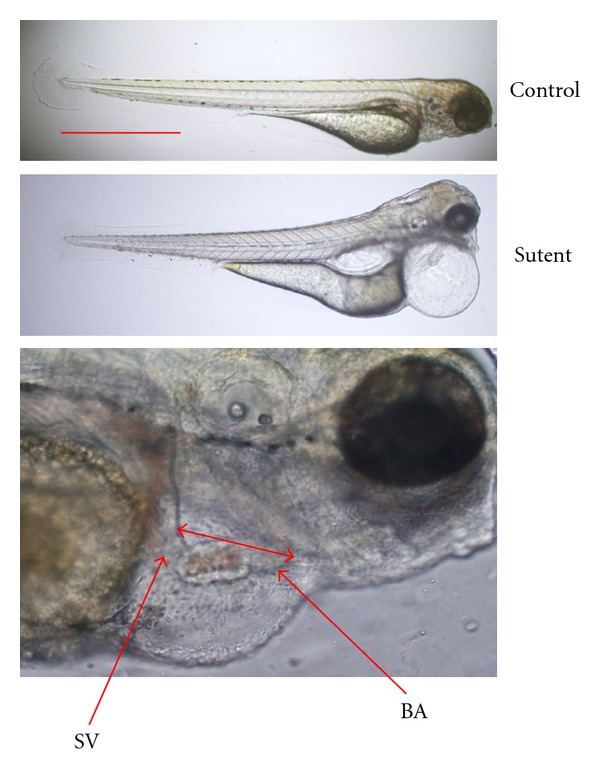
Sutent-induced cardiotoxicity in zebrafish embryos. Embryos at 5 dpf were treated with Sutent for 72 h followed by assessment of cardiac function. (a) Pericardial edema was observed after administration of Sutent compared to the control. The severity of pericardial edema was quantified by measuring the distance between sinus venosus (SV) and bulbus arteriosus (BA). (b) Embryo heart rate was decreased by treatment with Sutent in dose-dependent manner.

The zebrafish is a good model for studying cardiotoxicity. The cardiac function can be studied in zebrafish embryos through assessment of heart rate, heart morphology, cardiac myocytes number, and heart size [101]. Recently, we explored the cardiotoxicity of chemotherapeutic agents such as sunitinib malate (Sutent; SU11248; Pfizer). Sutent is a multitargeted tyrosine kinase inhibitor with antiangiogenic activity. It has been approved for first-line and adjuvant treatment of renal cell carcinoma. However, long-term angiogenesis inhibition would involve unwanted side effects, including cardiac and renal toxicity in patients with cancer [102]. Our study showed that Sutent deteriorates heart function through induction of pericardial edema and decrease in heart rate in zebrafish embryos (Figure 7).

## 11. Cerebral Hemorrhage Model

**Figure 8:**
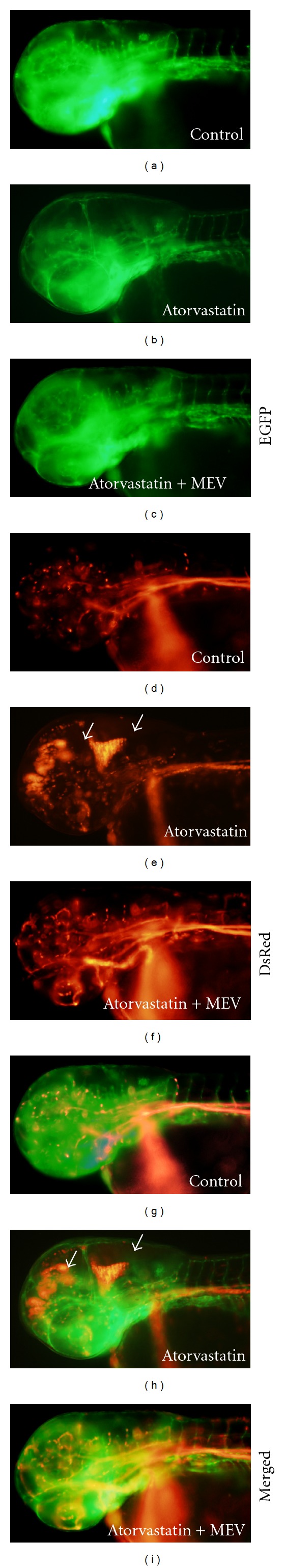
Atorvastatin-induced cerebral hemorrhage in zebrafish embryos. Tg(fli1:EGFP); Tg(gata1:dsred) homozygous double transgenic zebrafish embryos at 24 hpf were treated with atorvastatin alone or with atorvastatin and mevalonate (MEV) in combination for 24 h. Images showing blood vessels (EGFP, green) were superimposed on images showing blood flow (DsRed, red). Hemorrhage observed in the atorvastatin treatment group was prevented by cotreatment with MEV. Fluorescent microscopic images are at magnification 100x.

Cerebral hemorrhage, also known as hemorrhagic stroke, occurs when a blood vessel in the brain becomes weak and bursts, allowing blood to leak into the brain. Atorvastatin, a 3-hydroxy-3-methylglutaryl coenzyme-A (HMG-CoA) reductase inhibitor, reduces cholesterol, ameliorates, and vascular atherosclerosis and improves cardiovascular morbidity and mortality [103]. Pretreatment with atorvastatin significantly reduced infarct volume induced by permanent middle cerebral artery occlusion in animal studies [104]. Clinical studies showed patients with postischemic-stroke treatment with atorvastatin showed improving neurological recovery [105]. However, this beneficial effect is partly counteracted by an increased risk of hemorrhagic stroke [106]. Moreover, atorvastatin induced intracranial hemorrhages in wildtype fish [107] and induced cerebral hemorrhage in a zebrafish model (Figure 8), which offers an opportunity to screen cerebrovascular-protective compounds.

## 12. Dyslipidemia and Hyperlipidemia

The zebrafish model can be used in the study of lipid metabolism. The quenched fluorescent phospholipid substrate *N*-((6-(2,4-dinitrophenyl)amino)hexanoyl)-1-palmitoyl-2-BODIPY-FL-pentanoyl-*sn*-glycero-3-phosphoethanolamine (PED6) taken up by zebrafish larvae can fluoresce after cleavage by phospholipases in the intestine. It has been reported that this assay can be used to detect the fat-free (*ffr*) mutation, which likely results in disturbed lipid processing through impaired intestinal phospholipase activity [108] and reduced protease activity [109]. A research team led by Stoletov has developed a hypercholesterolemic (HCD) model in zebrafish utilizing a fluorescent cholesteryl ester to observe vascular lipid accumulation and fluorescent dextran in the endothelial cell layer disorganization after an HCD diet [110]. The reliability of the model was further supported by accumulation of macrophages, increased phospholipase A_2_ activity, and elevated levels of oxidized phosphatidylcholines in zebrafish fed an HCD diet compared to those fed a normal diet [110, 111]. Another research group led by Jin has demonstrated the antiatherosclerotic effect of turmeric and laurel aqueous extracts using this HCD model [112]. These disease models have proved to be highly relevant to human diseases and showed a number of conserved phenotype between zebrafish and human. Moreover, the difficulty of studying the atherogenic events in a temporal manner has been overcome due to its optical transparency.

## 13. Searching for Active Compounds from Natural Products

Many natural products exhibit a range of biological activity that is probably due to interaction of their complex chemical constituents with multiple targets in the body, which opens new avenues for therapy of disorders, with multifactorial etiopathogenesis such as neurodegeneration. The physiological complexity of zebrafish is similar to that of mammals, providing a suitable model for the study of human diseases as well as throughput drug screens. Using a whole organism as a model allows a more comprehensive and simultaneous analysis of the range of biological activity and toxicity of a chemical or multiple chemicals compared to an *in vitro* assay. Zebrafish embryos and early larvae are optically transparent, allowing screens with a measurable phenotypic readout using imaging microscopy for assessing pathological changes in Parkinson's disease, epilepsy, heart disease or cardiotoxicity, cerebral hemorrhage, and hyperlipidemia. This approach allows live and continuous observation on individuals which are often inapplicable in other *in vivo* models. More importantly, invasive approaches are often applied in these animal models, so reassessment of individuals may not be possible. For example, cerebral hemorrhage in rodent models was commonly done by intraparenchymal infusion of either autologous blood or bacterial collagenase. The hematoma size and location were evaluated with histologic analysis [113, 114]; hyperlipidemia in rodent models was achieved by feeding ApoE deficient mice with high fat diet for eight weeks and the atherosclerotic lesion was also observed by histology [115, 116]. Also, visual observation with imaging microscopy may require less technical skills and also far more convenient. For example, the evaluation of cardiotoxicity in zebrafish was determined by heart rate, heart morphology, cardiac myocyte number, and heart size. However, in rodent models, the heart function was often assessed by electrocardiogram or echocardiogram which requires intensive technical and labor input. Using rodent disease models for early stage drug screening may sound inapplicable since high-throughput studies are usually required. In addition, zebrafish model enables the observation of any pharmacological effect(s) on multiple targets underlying the pathway of a disease or a normal physiological process can be observed. The zebrafish model is, therefore, very suitable for identifying the off-target effects or multiple targets due to the holistic action of natural products.

## 14. Identifying Angiogenic Compounds from Natural Products

Angiogenesis is the establishment of the mature blood vessel network through expansion and remodeling of the vascular primordium. Blood vessel formation through angiogenesis involves the induction of new sprouts, coordinated and directed endothelial cell migration, proliferation, sprout fusion (anastomosis), and lumen formation [117]. Under normal conditions, tiny vessels do not increase in size or number, except in wound healing, embryonic development, and development of the corpus luteum. In fact, many diseases are associated with an imbalance in the regulation of angiogenesis, in which either excessive or insufficient blood vessel formation occurs.

To evaluate the angiogenic response in zebrafish, transgenic fish expressing green fluorescent protein (GFP) specifically in endothelial cells, for example, Tg(fli-1:EGFP) and Tg(fli-1:nEGFP), are recently used for rapid analysis of changed vasculature in live embryos in response to drugs [118]. In fact, zebrafish is an excellent animal model for the study of angiogenesis, with many antiangiogenic drugs eliciting responses similar to those in mammalian systems [119]. During the vasculature development, subintestinal vein vessels (SIVs) originate from the duct of Cuvier at 48 hpf and form a vascular basket in the yolk sac during the next 24 h. The angiogenic response was evaluated visually with respect to the following criteria: (1) the appearance of spikes or sprouts projecting from the subintestinal vessel basket or the lengthening of such spikes; (2) the extension of the basket into the yolk region with more than seven vertical branches within the basket [120]; (3) statistical increases in diameter compared to the medium control; (4) the ectopic growth of newly formed blood vessels from SIVs and increased numbers of SIVs in the endothelial cells [121]. Sprout formation was seen as the main characteristic in proangiogenesis [17, 120, 121]. Recently, we demonstrated the feasibility of drug screening in a zebrafish model and found the antiangiogenesis effect of a resveratrol derivative [52], indirubin [122], nobiletin [54], and sinensetin [51] as well as proangiogenesis effects of *Angelica sinensis* extract [123, 124], *Panax notoginseng* extract [125], and *Radix Astragali* extract [17].

Angiogenesis plays an important role in the development of human chronic inflammatory diseases, including cancer, psoriasis, rheumatoid arthritis, macular degeneration, and diabetes retinopathy [126, 127]. There is growing evidence that chronic inflammation and angiogenesis are codependent, involving increased cellular infiltration and proliferation as well as overlapping roles of regulatory growth factors and cytokines [126]. Persistent inflammation is linked with the progression of cancer, as proinflammatory cytokines are detected frequently in tumor tissue[128]. In rheumatoid arthritis, the formation of pannus [129], which is an inflammatory connective tissue mass rich in blood vessels, is apparently because angiogenic factors, such as VEGF, stimulate encephalitogenic T cells and induce more severe and prolonged encephalomyelitis [130]. Besides angiogenic factors, transcription factors such as NF-*κ*B plays a central role in the signaling of apoptosis and inflammation [131]. NF-*κ*B expression is associated with VEGF in the development and progression of tumorigenesis [132]. Signaling by the cyclooxygenase-2 (COX-2) downstream of NF-*κ*B may play a key role in the tumorigenesis of a variety of human malignancies by stimulating cell proliferation and angiogenesis [133]. Moreover, a recent study showed that chronic inflammation in benign prostatic hyperplasia causes an overexpression of COX-2, which induces the increased expression of Bcl-2 and VEGF [134].

Pharmacology of many anti-inflammatory drugs revealed at least some part of their efficacy is due to their antiangiogenic effect [126]. Tocotrienol, a member of the vitamin E family, possesses anticancer properties acting through regulating multiple signaling pathways, including anti-inflammation and antiangiogenesis [135]. The extract of *Physalis angulate* shows antimetastatic and antiangiogenic activity in human oral squamous carcinoma and human umbilical vein endothelial cells, probably due to its anti-inflammatory properties [136]. Indirubin inhibits inflammatory reactions by suppressing the production of interferon-*γ* and interleukin-6, which is a well-known inflammatory cytokine [137]. Interestingly, it also displays antiangiogenic activity by inducing HUVEC apoptosis and cell-cycle arrest at the G0/G1 phase [122]. Resveratrol and its derivative exert antiangiogenic and vascular-disrupting effects in zebrafish through downregulation of VEGFR2 and cell-cycle modulation [52]. The anti-inflammatory property of resveratrol is reported to prevent an increase in the levels of serum amyloid A, tumor necrosis factor-*α*, interleukin (IL-6), IL-1*β*, and nuclear transcription factor-*κ*B in colitis-associated disease [138].

Angiogenesis deficiencies are associated with numerous human cardiovascular and cerebrovascular diseases (e.g., ischemic cardiac and cerebral problems). Our previous discovery of a pro-angiogenic herb called *Angelica sinensis* by zebrafish assay leading to development of a wound healing formulation for diabetic foot ulcer patients [124, 139]. Our recent work presented, for the first time, that a chemical-induced blood vessel loss in zebrafish *in vivo* could mimic angiogenesis deficiencies associated with human disease conditions and be used to identify pro-angiogenic agents. VEGFR tyrosine kinase inhibitor II (VRI), a pyridinyl-anthranilamide compound that displays antiangiogenic properties, strongly inhibits the kinase activities of both VEGF receptor 1 and 2. Treatment of the zebrafish with VRI induces significant blood vessel loss in ISV (intersegmental vessels) and DLAV (dorsal longitudinal anastomotic vessels). For instance, we identified a polysaccharide fraction (50000 D < MW and DM < 0.1 *μ*m) isolated from Astragali Radix partially restores the chemical-induced blood vessel loss in the zebrafish model [140]. This is also the first study to prove the concept of screening the bioactivity of polysaccharides in live zebrafish, whose drug metabolism systems were shown recently to have a high degree of functional similarity to that of mammals. Since polysaccharides isolated from natural products usually undergo the enzymatic breakdown of the sugar moiety in the cells of the gastrointestinal mucosa, or by enzymes secreted by the colon flora, to become active metabolites after oral consumption by humans, the study of the bioactivity of the polysaccharides required the development of an *in vivo* assay equipped with mammalian-equivalent drug metabolism systems. Our findings provide insight into a new angiogenesis deficiency zebrafish model for screening vascular regenerative agents as well as the important roles of various substances from Chinese medicines for the treatment of various pathological conditions associated with deficient angiogenesis, such as ageing, stroke, ulcers, and cardiovascular diseases [140, 141].

## 15. Identifying Anti-Parkinson's Disease Compounds from Natural Products

PD patients usually suffer primarily from the death of dopaminergic (DA) neurons in the substantia nigra. Recent research in the pharmacotherapy of PD has identified numerous agents for the symptomatic control of motor impairments, but none is able to prevent, slow, or halt the progression of the disease [142]. The main obstacle to developing neuroprotective therapies is our limited understanding of the key molecular events that provoke neurodegeneration. Earlier studies highlighted the pathological involvement of oxidative stress, neuroinflammation, excitotoxicity, and apoptosis in neurodegenerative diseases [143]. Because PD, as well as other neurodegenerative disorders, usually has multifactorial etiopathogenesis, multiple drug therapy is required to address the varied pathological aspects [144]. Multiple drug strategy has been the essence of the rationales used for formulating traditional Chinese medicines (TCMs) for thousands of years. TCMs contain a mixture of chemical components from a single herb or a combination of several herbs and thus versatile functions and possess great potential in the multitarget approach for improved treatment of complicated diseases, such as PD.

By combining whole mount immunostaining and a behavioural screen, we have identified the neuroprotective activity of a few TCMs, including Fructus *Alpinia oxyphylla *extract (AOE) and *Eriocaulon buergerianum* extract (EBE) [75, 145]. Recently, increasing evidence suggests the beneficial effects of Fructus AOE on various neurodegenerative diseases. Treatment with aqueous AOE attenuated the death of cortical astrocytes induced by amyloid-*β* (A*β*) *in vitro*, prevented ischemia-induced learning disability, and rescued hippocampal CA1 neurons from lethal ischemic damage in mice [146]. Treatment with the ethanolic AOE in the presence of glutamate significantly enhanced viability and reduced apoptosis in a cortical neuron culture [147]. We found that ethanolic AOE prevented and restored 6-OHDA-induced DA neurodegeneration and attenuated the deficit of locomotor activity in zebrafish [75]. In addition, the aquatic plant EB (Gujingcao) is a TCM with anti-inflammatory and antimicrobial properties [148]. In the Chinese Pharmacopoeia (2005), the capitulum of EB is one of the most frequently used Chinese medicinal herbs, with flavonoids, volatile oils, anthraquinone, naphthopyranones, protocatechuic acid, and c-tocopheryl acetate being the bioactive constituents [149]. Flavonoids such as patuletin hispidulin, quercetin, quercetagetin, and quercetagetin derivatives and volatile oil such as palmitic acid, (Z,Z)-9,12-octacosane-dienoic acid are the two major classes of chemicals in EB [148]. EB demonstrates significant therapeutic effects on headache, toothache, nasosinusitis, night blindness, glaucoma, retinochoroiditis, conjunctivitis, and other eye diseases [150]. The results of our study suggested that EBE has profound neuroprotective activity in zebrafish, including the dose-dependent recovery of DA neuron loss caused by 6-OHDA *in vivo* and inhibition of the 6-OHDA-induced decrease of total movement distance in zebrafish [145]. We found that quercetin was one of the active neuroprotective constituents in EBE [28]. All these groundwork warrants further study of how the interaction of multiple components in these natural products elicits neuroprotection.

## 16. Conclusions

Zebrafish offers interesting possibilities for the simultaneous assessment of efficacy and toxicity of target compounds, which is not easily addressed with current rodent models. With its physiological similarities to human, many disease models could be established for identifying the off-target and the targeted effects of target compounds. More importantly, it allows integrative studies of transcriptomics and proteomics for identifying drug metabolic pathways and known or novel molecular targets involved in the biological response of zebrafish to drug treatment.

## Figures and Tables

**Table 1:** Discrepancies and similarities of the effect of drugs in human and zebrafish.

Area of evaluation in zebrafish	Test compounds	Proportion of drugs with expected effects (%)	Reference
Inhibition of hERG or QTc prolongation	Study 1: Amiodarone, bepridil, cisapride, haloperidol, pimozide, procainamide, D,L-sotalol, terfenadine, thioridazine	All compounds, except for procainamide	[3]
	Study 2: Negative controls: amoxicillin, aspirin Positive controls: chlorpromazine, cisapride, cromakalim, isoprenaline, moxifloxacin, nicotine, verapamil	7 out of 9 compounds, including negative controls	[1]

Visual safety or optomotor response	Study 1: 27 compounds, including 19 with positive and 8 with negative effects on inhibition of optomotor response	About 70% in overall showed the predicted drug effects.	[4]
	Study 2: Negative control: aspirin Positive controls: chloroquine, chlorpromazine, diazepam, nicotine, ouabain, phenytoin, atropine, lithium	7 out of 9 compounds including negative control	[1]

Seizure liability	25 drugs including 17 positive and 8 negative controls	72% in overall	[5]

Gut contraction	Negative controls: aspirin and moxifloxacin Positive controls: amoxicillin, chlorpromazine, cisapride, cromakalim, isoprenaline, nicotine, nitrendipine, and verapamil	5 out of 10 compounds including negative controls	[1]

**Table 2:** Potential marker genes for PD.

Gene	Function	Assessment method	Reference
Tyrosine hydroxylase (TH)	Catalytic conversion of the amino acid L-tyrosine to dihydroxyphenylalanine	Immunostaining, locomotion behaviour test	[28, 64, 66]
Dopamine transporter (DAT)	Membrane-spanning protein for pumping neurotransmitter DA back into cytosol from the synaptic region	Whole mount *in situ* hybridization (WISH), swimming behaviour	[65]
Vesicular monoamine transporter 2 (VMAT2)	Integral membrane protein for transporting neurotransmitter carrying monoamine structure, for example, dopamine and norepinephrine from cellular cytosol into synaptic vesicles	Visualization in VMAT2: GFP transgenic fish	[67]
MAO-B	Catalytic oxidation of monoamines	Monoamine oxidase enzyme histochemistry	[66]
PARKIN (PARK2)	Gene knockdown leads to complex I deficiency and dopaminergic neuronal cell loss	WISH, whole-mount antibody immunofluorescence, behaviour analysis	[68]
LRRK2	Genetic mutant caused loss of DA neuron and locomotive defect	WISH, swimming behaviour	[69]

**Table 3:** Potential marker genes for epilepsy.

Function	Gene	Assessment method	References
Transcription factor	c-Fos	Immunohistochemistry, *In situ *hybridization, real-time PCR	[85]
	c-Jun	Electrophoretic mobility-shift assay	[86]
	CREB	Real-time PCR, northern blot	[87]
	Zac 1	Immunohistochemistry, *In situ *hybridization	[88]
Receptor	NMDAR1	Immunohistochemistry, Western blot	[89]
	GABA(A)-receptor delta	Immunohistochemistry	[81]
Ion channel	Kv1.2 and Kv4.2	*In situ *hybridization	[90]
Transporter	GLUT1 and GLUT3	*In situ *hybridization, Western blot	[91]

**Table 4:** Potential biomarkers for human heart disease.

Gene	Function	Assessment method	Associated cardiovascular disease
Troponin T	Myocardial contraction	ELISA	Congestive heart failure [96, 97]
Heart fatty acid binding protein	Carrier proteins for fatty acids and other lipophilic substances, such as eicosanoids and retinoids	ELISA	Congestive heart failure [96, 97]
Myosin light chain-I	Myocardial contraction	ELISA	Congestive heart failure [96, 100]
Creatine kinase MB	Energy metabolism	ELISA	Congestive heart failure [96]
Cardiotrophin-1	Response to stress and humoral factors such as angiotensin II	ELISA	Hypertension [98]
Endothelin-1	Potent endothelium-derived vasoconstrictor peptide	Radioimmunoassay	Heart failure [99]
